# Endothelial Dysfunction: From Pathophysiology to Novel Therapeutic Approaches 2.0

**DOI:** 10.3390/biomedicines13112639

**Published:** 2025-10-28

**Authors:** Hee Kyoung Joo, Byeong Hwa Jeon

**Affiliations:** 1 Department of Physiology, College of Medicine, Chungnam National University, Daejeon 35015, Republic of Korea; 2 Research Institute of Medical Sciences, College of Medicine, Chungnam National University, Daejeon 35015, Republic of Korea; 3 Department of Medical Science, College of Medicine, Chungnam National University, Daejeon, 35015, Republic of Korea

Endothelial dysfunction (ED) is a multifactorial pathological process characterized by the loss of the endothelial physiological function, which is essential for maintaining vascular homeostasis [1]. ED results from chronic exposure to stressors, such as inflammation, oxidative stress, hypertension, and hypercholesterolemia [2,3,4,5]. This resulting endothelial pathological cascade involves vessel wall damage, smooth muscle proliferation, and immune cell infiltration. ED acts as a common pathophysiological mechanism across a broad spectrum of clinical diseases, including post-COVID syndrome, osteonecrosis (ONFH), and cardiovascular diseases (CVD). Recognizing this shared basis of compromised endothelial health shifts the therapeutic focus toward targeting the common vascular dysfunction.

The articles in this Special Issue compellingly illustrate ED’s central role, highlighting that the health of the endothelium is a fundamental determinant of overall health. The contributions illuminate shared pathological drivers by grouping them into two core, mechanistically interconnected themes: Ischemia and Oxidative Stress/Inflammation.

The most common consequence of ED is impaired blood flow and local tissue perfusion, leading to tissue ischemia. Ladek et al. provide compelling evidence linking local deoxygenation and post-exertional malaise in post-COVID syndrome to underlying vascular issues, suggesting that ED or ischemia may be a key driver of post-COVID symptoms. Their work validates non-invasive near-infrared regional spectroscopy (NIRS) as a promising non-invasive tool for assessing these microcirculatory deficits [6]. However, the limited sample size and the cross-sectional nature of the data establish a strong association but do not confirm causality and require longitudinal studies. The connection between ED and osteonecrosis of the femoral head (ONFH) is a crucial area of research. ONFH is fundamentally a debilitating disease caused by insufficient blood flow to the subchondral bone [7,8]. A comprehensive review by Shao et al. details ED as a central cause through mechanisms of coagulopathy and impaired angiogenesis [9]. This review offers new perspectives on treatment strategies by suggesting that targeting the endothelium could prevent bone death and promote healing. A key limitation here is the reliance on animal models and a lack of human data (human ONFH specimens); another limitation is etiological heterogeneity of ONFH (glucocorticoid use, alcohol abuse, etc.); future research must clarify if the inflammatory cascades driven by these distinct risk factors truly converge on a unified vascular pathology or require risk factor-specific therapeutic targeting for osteonecrosis.

Multiple studies show how elevated oxidative stress and chronic inflammation directly damage the endothelium, often triggering cellular death. Homocysteine (Hcy) is an amino acid produced during the metabolism of methionine, an essential amino acid. Under normal conditions, Hcy is rapidly converted into other useful substances, such as cysteine, with the help of various vitamins (folate, B6, B12) [10]. Hyperhomocysteinemia is linked to a wide range of CVD, including atherosclerosis, hypertension, and dementia [11,12,13,14]. A study with Shi et al. showed that Hcy toxicity causes endothelial cell death through apoptosis and autophagy. They found that this toxicity results from elevated reactive oxygen species (ROS), mitochondrial dysfunction, and abnormal iron metabolism in a preclinical in vitro model (HUVECs) [15]. This in vitro model, however, introduces inherent translational limitations, demanding further validation in complex in vivo animal and human models before any therapeutic targeting can be recommended. Carotenoids are the most widely distributed pigments in nature, found in a variety of fruits and vegetables [16,17]. Among carotenoids, capsanthin is a lipophilic red pigment responsible for the red color of paprika fruits [18,19]. Kim et al. found that capsanthin has been shown to reduce inflammatory markers and inhibit the activation of key inflammatory pathways in endothelial cells. Furthermore, dietary capsanthin can significantly inhibit the formation of atherosclerotic plaques in the aorta of ApoE^−/−^ mice, suggesting that a diet rich in capsanthin could potentially prevent atherosclerosis [20]. Despite the optimism generated by these animal model findings, these preclinical results are insufficient for therapeutic recommendations, requiring essential clinical studies for validation. Selenium is a crucial trace mineral for human health, primarily because it is a key component of selenoproteins, which are essential for various metabolic processes [21,22]. A review by Dabravolski et al. comprehensively details the molecular basis of selenium’s athero-protective activities, covering its role in vascular calcification, apoptosis, autophagy, and endothelial dysfunction [23]. Recent research highlights the complex role of selenium in obesity, a growing global health concern [24]. Selenium and its compounds demonstrate significant antioxidant activity and are important for the nutritional evaluation of obese children. The balance of selenium intake, retention, and metabolism is thus a vital aspect of health, reflecting the intricate interactions between diet, oxidative stress, and obesity. While selenium regulates redox homeostasis and ED [21,22,23,24], the most significant challenge lies in the inconsistent outcomes of human randomized controlled trials due to a narrow therapeutic window and U-shaped risk curve [23]. Current clinical data remain insufficient to broadly recommend selenium supplementation without personalized diagnostics to confirm true functional deficiency.

This Special Issue provides a strong foundation for future study. The field must now shift toward personized medicine by identifying specific biomarkers that can predict which patients are most vulnerable to ED. Promising classes include circulating endothelial cells, which reflect vascular damage, and microRNAs, which regulate gene expression in the endothelium. Further development of non-invasive tools like NIRS for tissue oxygenation and high-resolution ultrasound for real-time flow-mediated dilation is crucial for early detection and monitoring of microcirculatory deficits. The mechanistic parallels across the studies linking inflammation/oxidative stress (Hcy, nutritional) to ischemia (post-COVID, ONFH) point toward clear therapeutic paths. Realistic strategies include moving selective anti-inflammatory agents (targeting NF-κB or specific cytokines) or antioxidant selenoproteins into clinical trials for conditions where ED is a primary driver of tissue damage. By building on these insights, the scientific community can shift from treating downstream symptoms to protecting the vascular endothelium.
